# Opening the AI Black Box: Distilling Machine-Learned Algorithms into Code

**DOI:** 10.3390/e26121046

**Published:** 2024-12-02

**Authors:** Eric J. Michaud, Isaac Liao, Vedang Lad, Ziming Liu, Anish Mudide, Chloe Loughridge, Zifan Carl Guo, Tara Rezaei Kheirkhah, Mateja Vukelić, Max Tegmark

**Affiliations:** 1 Department of Physics, Massachusetts Institute of Technology, Cambridge, MA 02139, USA; ericjm@mit.edu (E.J.M.); zmliu@mit.edu (Z.L.); 2 Institute for Artificial Intelligence and Fundamental Interactions, Cambridge, MA 02139, USA; 3 Department of Electrical Engineering and Computer Science, Massachusetts Institute of Technology, Cambridge, MA 02139, USA; iliao@mit.edu (I.L.); vedang@mit.edu (V.L.); amudide@mit.edu (A.M.); carlguo@mit.edu (Z.C.G.); tarark@mit.edu (T.R.K.); mvukelic@mit.edu (M.V.); 4 Harvard College, Cambridge, MA 02138, USA; cloughridge@college.harvard.edu

**Keywords:** mechanistic interpretability, program synthesis

## Abstract

Can we turn AI black boxes into code? Although this mission sounds extremely challenging, we show that it is not entirely impossible by presenting a proof-of-concept method, MIPS, that can synthesize programs based on the automated mechanistic interpretability of neural networks trained to perform the desired task, auto-distilling the learned algorithm into Python code. We test MIPS on a benchmark of 62 algorithmic tasks that can be learned by an RNN and find it highly complementary to GPT-4: MIPS solves 32 of them, including 13 that are not solved by GPT-4 (which also solves 30). MIPS uses an integer autoencoder to convert the RNN into a finite state machine, then applies Boolean or integer symbolic regression to capture the learned algorithm. As opposed to large language models, this program synthesis technique makes no use of (and is therefore not limited by) human training data such as algorithms and code from GitHub. We discuss opportunities and challenges for scaling up this approach to make machine-learned models more interpretable and trustworthy.

## 1. Introduction

Machine-learned algorithms now outperform traditional human-discovered algorithms on many tasks, from translation to general-purpose verbal reasoning. These learned algorithms tend to be black box neural networks, and we typically lack a full understanding of how they work. This is part of the reason why many leading AI researchers and business leaders have warned that the seemingly unstoppable race toward ever-more-powerful AI could end badly for humanity [1]. Regardless of one’s views on this controversial matter, there is broad agreement that improved reliability and trustworthiness are valuable as AI becomes ever more capable [2,3].

Our research question is as follows: How can we ensure that AI models are truly implementing their intended functions? Without formal proof, we can never be certain. We propose transforming AI models into programs that can be formally verified. Yet formal verification, the gold standard for reliability, is widely viewed as unattainable for powerful AI systems because

1. Neural networks seem too messy to formally verify;
2. Mechanistic interpretability converting learned powerful neural network algorithms into formally verifiable code seems too hard and labor-intensive.

Our paper aims to inject optimism into this field by taking a small but nontrivial step toward showing that automatic mechanistic interpretability (AutoMI) is not impossible, where AutoMI means black box models can be turned into programs automatically without human inspection. Specifically, we present a proof-of-concept method, MIPS (mechanistic-interpretability-based program synthesis), which can distill simple learned algorithms from neural networks into Python code, for small-scale algorithmic tasks. The main goal of this paper is not to present a method that fully solves AutoMI, but to demonstrate progress toward AutoMI with a simple proof of concept. The rest of this paper is organized as follows. After reviewing prior work in Section 2, we present our method in Section 3, test it on a benchmark in Section 4 and summarize our conclusions in Section 5.

## 2. Related Work

Program synthesis is a venerable field dating back to Alonzo Church in 1957; Zhou and Ding [4] and Odena et al. [5] provide recent reviews of the field. Large language models (LLMs) have become increasingly good at writing code based on verbal problem descriptions or auto-complete. We instead study the common alternative problem setting known as “programming by example” (PBE), where the desired program is specified by giving examples of input–output pairs [6]. The aforementioned papers review a wide variety of program synthesis methods, many of which involve some form of search over a space of possible programs. LLMs that synthesize code directly have recently become quite competitive with such search-based approaches [7]. Our work provides an alternative search-free approach where the program learning happens during neural network training rather than execution.

Our work builds on the recent progress in mechanistic interpretability (MI) of neural networks [8,9,10,11]. Much MI work has tried to understand how neural networks represent various types of information, e.g., geographic information [12,13,14], truth [15,16] and the state of board games [17,18,19]. Another major MI thrust has been to understand how neural networks perform algorithmic tasks, e.g., modular arithmetic [20,21,22,23] and other group operations [24], greater than [25], and greatest common divisor [26]. The key step of mechanistic interpretability is to look into discovering structures in hidden representations. In this paper, we manage to discover bit representations, integer representations, and clusters (finite state machines).

Whereas Lindner et al. [27] automatically convert traditional code into a neural network, we aim to do the opposite, as was also recently demonstrated in Friedman et al. [28]. One direction in automating mechanistic interpretability uses LLMs to label internal units of neural networks such as neurons [29] and features discovered by sparse autoencoders [30,31]. Another recent effort in automating MI involves automatically identifying which internal units causally influence each other and the network output for a given set of inputs [32,33,34]. However, these methods do not automatically generate pseudocode or give a description of *how* the states of downstream units are computed from upstream units, which we aim to do in this work.

In this work, we focus on automating mechanistic interpretability for recurrent neural networks (RNNs), building on the rich existing literature on interpreting RNN internals [35,36] and on extracting finite state machines from trained RNNs [36,37,38,39,40,41]. In our work, we seek exceptionally simple descriptions of RNNs by factoring network hidden states into discrete variables and representing state transitions with symbolic formulae.

## 3. MIPS, Our Program Synthesis Algorithm

**Figure 1:**
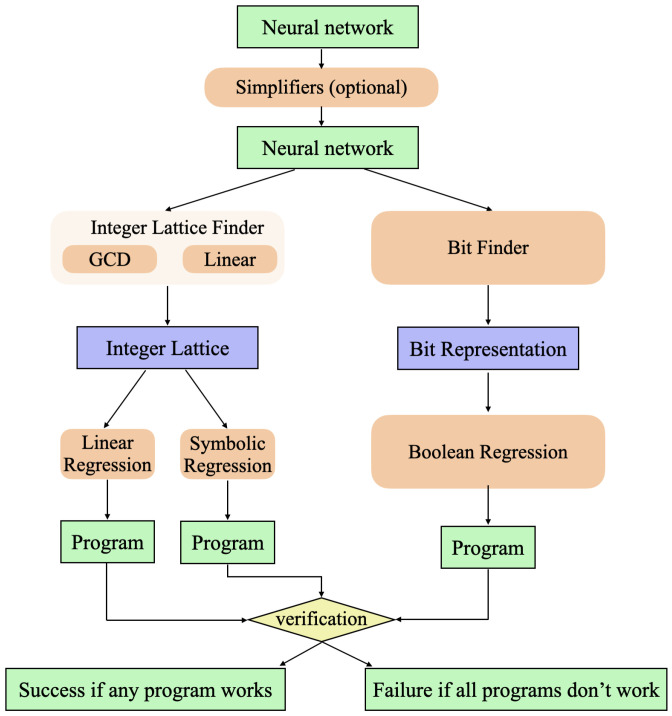
The pipeline of our program synthesis method. MIPS relies on discovering integer representations and bit representations of hidden states, which enable regression methods to figure out the exact symbolic relations between input, hidden, and output states.

As summarized in Figure 1, MIPS involves the following key steps.

1. Neural network training;
2. Neural network simplification;
3. Finite state machine extraction;
4. Symbolic regression.

Step 1 is to train a black box neural network to learn an algorithm that performs the desired task. In this paper, we use a recurrent neural network (RNN) of the general form

(1) $$\begin{array}{ccc} h_{i} & = & f(h_{i-1},x_{i}), \end{array}$$

(2) $$\begin{array}{ccc} y_{i} & = & g(h_{i}), \end{array}$$

that maps input vectors $x_{i}$ into output vectors $y_{i}$ via hidden states $h_{i}$. The RNN is defined by the two functions *f* and *g*, which are implemented as feed-forward neural networks (MLPs) to allow more model expressivity than a vanilla RNN. The techniques described below can also be applied to more general neural network architectures.

Step 2 attempts to automatically simplify the learned neural network without reducing its accuracy. Steps 3 and 4 automatically distill this simplified learned algorithm into Python code. When the training data are discrete (consisting of, say, text tokens, integers, or pixel colors), the neural network will be a *finite state machine*: the activation vectors for each of its neuron layers define finite sets and the entire working of the network can be defined by look-up tables specifying the update rules for each layer. For our RNN, this means that the space of hidden states h is discrete, so the functions *f* and *g* can be defined by lookup tables. As we will see below, the number of hidden states that MIPS needs to keep track of can often be greatly reduced by clustering them, corresponding to learned representations. After this, the geometry of the cluster centers in the hidden space often reveals that they form either an incomplete multidimensional lattice whose points represent integer tuples, or a set whose cardinality is a power of two, whose points represent Boolean tuples. In both of these cases, the aforementioned lookup tables simply specify integer or Boolean functions, which MIPS attempts to discover via symbolic regression. Below, we present an *integer autoencoder* and a *Boolean autoencoder* to discover such integer/Boolean representations from arbitrary point sets.

We will now describe each of the three steps of MIPS in greater detail.

### 3.1. AutoML Optimizing for Simplicity

We wish to find the *simplest* RNN that can learn our task, to facilitate subsequent discovery of the algorithm that it has learned. We therefore implement an AutoML-style neural architecture search that tries to minimize network size while achieving perfect test accuracy. This search space is defined by a vector p of five main architecture hyperparameters: the five integers $p=(n,w_{f},d_{f},w_{g},d_{g})$ corresponding to the dimensionality of hidden state h, the width and depth of the *f*-network, and the width and depth of the *g*-network, respectively. Both the *f*- and *g*-networks have a linear final layer and ReLU activation functions for all previous layers. The hidden state $h_{0}$ is initialized to zero.

To define the parameter search space, we define ranges for each parameter. For all tasks, we use n∈{1,2,…,128}, $w_{f}\in \left\{1,2,\ldots ,256\right\}$, $d_{f}\in \left\{1,2,3\right\}$, $w_{g}\in \left\{1,2,\ldots ,256\right\}$ and $d_{g}\in \left\{1,2,3\right\}$, so the total search space consists of 128×256×3×256×3 = 75,497,472 hyperparameter vectors $p_{i}$. We order this search space by imposing a strict ordering on the importance of minimizing each hyperparameter–lower $d_{g}$ is strictly more important than lower $d_{f}$, which is strictly more important than lower *n*, which is strictly more important than lower $w_{g}$, which is strictly more important than lower $w_{f}$. We aim to find the hyperparameter vector (integer 5-tuple) $p_{i}$ in the search space that has the lowest rank *i* under this ordering.

We search the space in the following simple manner. We first start at index i= 65,536, which corresponds to parameters (1,1,2,1,1). For each parameter tuple, we train networks using five different seeds. We use the loss function $\ell \left(x,y\right)=\frac{1}{2}\log\left[1+{\left(x-y\right)}^{2}\right]$, finding that it leads to more stable training than using vanilla MSE loss. We train for either 10,000 or 20,000 steps, depending on the task, using the Adam optimizer, a learning rate of $10^{-3}$, and batch size 4096. The test accuracy is evaluated with a batch of 65536 samples. If no networks achieve 100% test accuracy (on any test batch), we increase *i* by $2^{1/4}$. We proceed in this manner until we find a network where one of the seeds achieves perfect test accuracy or until the full range is exhausted. If we find a working network on this upwards sweep, we then perform a binary search using the interval halving method, starting from the successful *i*, to find the lowest *i* where at least one seed achieves perfect test accuracy.

### 3.2. Auto-Simplification

After finding a minimal neural network architecture that can solve a task, the resulting neural network weights typically seem random and un-interpretable. This is because there exist symmetry transformations of the weights that leave the overall input–output behavior of the neural network unchanged. The random initialization of the network has therefore caused random symmetry transformations to be applied to the weights. In other words, the learned network belongs to an equivalence class of neural networks with identical behavior and performance, corresponding to a submanifold of the parameter space. We exploit these symmetry transformations to simplify the neural network into a *normal form*, which in a sense is the simplest member of its equivalence class. Conversion of objects into a normal/standard form is a common concept in mathematics and physics (for example, conjunctive normal form, wavefunction normalization, reduced row echelon form, and gauge fixing).

Two of our simplification strategies below exploit a symmetry of the RNN hidden state space h. We can always write the MLP *g* in the form g(h)=G(Uh+c) for some function *G*. So if *f* is affine, i.e., of the form f(h,x)=Wh+Vx+b, then the symmetry transformation.

$W^{\prime }\equiv AWA^{-1}$, $V^{\prime }=AV$, $U^{\prime }=UA^{-1}$, $h^{\prime }\equiv Ah$, $b^{\prime }=Ab$ keeps the RNN in the same form:

(3) $$\begin{array}{ccc} h_{i}^{\prime } & = & Ah_{i}=AWA^{-1}Ah_{i-1}+AVx_{i}+Ab\\ & = & W^{\prime -1}h_{i-1}^{\prime }+V^{\prime }x_{i}+b^{\prime }, \end{array}$$

(4) $$\begin{array}{ccc} y_{i} & = & G\left(Uh_{i}+c\right)=G\left(UA^{-1}h_{i}^{\prime }+c\right)\\ & = & G(U^{\prime }h_{i}^{\prime }+c). \end{array}$$

We think of neural networks as nails, which can be hit by various auto-normalization hammers. Each hammer is an algorithm that applies transformations to the weights to remove degrees of freedom caused by extra symmetries or cleans the neural network up in some other way. In this section, we describe five normalizers we use to simplify our trained networks, termed “Whitening”, “Jordan normal form”, “Toeplitz”, “De-bias”, and “Quantization”. For every neural network, we always apply this sequence of normalizers in that specific order, for consistency. We describe them below and provide additional details about them in the Appendix D.

1. Whitening: Just as we normalize input data to use for training neural networks, we would like activations in the hidden state space $h_{i}$ to be normalized. To ensure normalization in all directions, we feed the training dataset into the RNN, collect all the hidden states, compute the uncentered covariance matrix C, and then apply a whitening transform $h\mapsto C^{-1/2}h$ to the hidden state space so that its new covariance becomes the identity matrix. This operation exists purely to provide better numerical stability to the next step.
2. Jordan normal form: When the function *g* is affine, we can apply the aforementioned symmetry transformation to try to diagonalize W, so that none of the hidden state dimensions interact with one another. Unfortunately, not all matrices W can be diagonalized, so we use a generalized alternative: the Jordan normal form, which allows elements of the superdiagonal to be either zero or one. To eliminate complex numbers, we also apply 2×2 unitary transformations to eigenvectors corresponding to conjugate pairs of complex eigenvalues afterward. The aforementioned whitening is now ruined, but it helped make the Jordan normal form calculation more numerically stable.
3. Toeplitz: Once W is in a Jordan normal form, we divide it up into Jordan blocks and apply upper-triangular Toeplitz transformations to the dimensions belonging to each Jordan block. There is now an additional symmetry, corresponding to multiplying each Jordan block by an upper triangular Toeplitz matrix, and we exploit the Toeplitz matrix that maximally simplifies the aforementioned V-matrix.
4. De-bias: Sometimes W is not full rank, and b has a component in the direction of the nullspace. In this case, the component can be removed, and the bias c can be adjusted to compensate.
5. Quantization: After applying all the previous normalizers, many of the weights may have become close to integers, but not exactly due to machine precision and training imperfections. Sometimes, depending on the task, all of the weights can become integers. We therefore round any weights that are within ϵ≡0.01 of an integer to that integer.

### 3.3. Boolean and Integer Autoencoders

As mentioned, our goal is to convert a trained recurrent neural network (RNN) into a maximally simple (Python) program that produces equivalent input–output behavior. This means that if the RNN has 100% accuracy for a given dataset, so should the program, with the added benefit of being more interpretable, precise, and verifiable.

Once trained/written, the greatest difference between a neural network and a program implementing the same finite state machine is that the former is fuzzy and continuous, while the latter is precise and discrete. To convert a neural network to a program, some discretization (“defuzzification”) process is needed to extract precise information from seemingly noisy representations. Fortunately, mechanistic interpretability research has shown that neural networks tend to learn meaningful, structured knowledge representations for algorithmic tasks [20,21]. Previous interpretability efforts typically involved case-by-case manual inspection and only gained algorithmic understanding at the level of pseudocode at best. We tackle this more ambitious question: Can we create an automated method that distills the learned representation and associated algorithms into an equivalent (Python) program?

**Figure 2:**
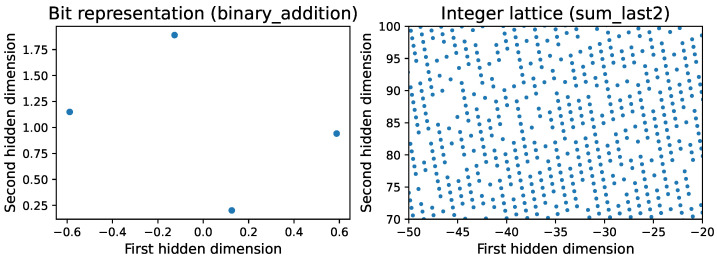
These hidden structures can be turned into discrete representations. Left: bitstring addition, corresponding to 2 bits: the output bit and the carry bit. Right: Sum_Last2 task, 2D lattice corresponding to two integers.

Since the tasks in our benchmark involve bits and integers, which are already discrete, the only non-discrete parts in a recurrent neural network are its hidden representations. Here, we show two cases when hidden states can be discretized: they are (1) a bit representation or (2) a (typically incomplete) integer lattice. Generalizing to the mixed case of bits and integers is straightforward. Figure 2 shows all hidden state activation vectors $h_{i}$ for all steps with all training examples for two of our tasks. The left panel shows that the $10^{4}$ points $h_{i}$ form $2^{2}=4$ tight clusters, which we interpret as representing two bits. The right panel reveals that the points $h_{i}$ form an incomplete 2D lattice that we interpret as secretly representing a pair of integers.

#### 3.3.1. Bit Representations

The hidden states for the two bits in Figure 2 are seen to form a parallelogram. More generally, we find that hidden states encode *b* bits as $2^{b}$ clusters, which in some cases form *b*-dimensional parallelograms and in other cases look more random. Our algorithm tries all $(2^{b})!$ possible assignments of the $2^{b}$ clusters to bitstrings of length *b* and selects the assignment that minimizes the length of the resulting Python program.

#### 3.3.2. Integer Lattice

As seen in Figure 2, the learned representation of an integer lattice tends to be both non-square (deformed by a random affine transformation) and sparse (since not all integer tuplets occur during training). We thus face the following problem: given (possibly sparse) samples of points $h_{i}$ from an *n*-dimensional lattice, how can we reconstruct the integer lattice in the sense that we figure out which integer tuple each lattice point represents? We call the solution an *integer autoencoder* since it compresses any point set into a set of integer tuples from which the original points can be at least approximately recovered as $h_{i}=Ak_{i}+b$, where A is a matrix and b is a vector that defines the affine transformation and a set of integer vectors $k_{i}$.

In the Appendix A, we present a solution that we call the *GCD lattice finder*. For the special case n=1, its core idea is to compute the greatest common denominator of pairwise separations: for example, for the points {1.7,3.2,6.2,7.7...}, all point separations are divisible by A=1.5, from which one infers that b=0.2 and the lattice can be rewritten as 1.5×{1, 2, 4, 5}+0.2. For multidimensional lattices, our algorithm uses the GCD of ratios of generalized cell volumes to infer the directions and lengths of the lattice vectors that form the columns of A.

For the special case where the MLP defining the function *f* is affine or can be accurately approximated as affine, we use a simpler method we term the *Linear lattice finder*, also described in Appendix B. Here, the idea is to exploit the fact that the lattice is simply an affine transformation of a regular integer lattice (the input data), so we can simply “read off" the desired lattice basis vectors from this affine transformation.

#### 3.3.3. Symbolic Regression

Once the hidden states $h_{i}$ have been successfully mapped to Boolean or integer tuples as described above, the functions *f* and *g* that specify the learned RNN can be re-expressed as lookup tables, showing their Boolean/integer output tuple for each Boolean/integer input tuple. All that remains is now symbolic regression, i.e., discovering the simplest possible symbolic formulae that define *f* and *g*.

#### 3.3.4. Boolean Regression

In the case where a function maps bits to a bit, our algorithm determines the following set of correct Boolean formulae and then returns the shortest one. The first candidate formula is the function written in disjunctive normal form, which is always possible. If the Boolean function is symmetric, i.e., invariant under all permutations of its arguments, then we also write it as an integer function of its bit sum.

#### 3.3.5. Integer Regression

In the case when a function maps integers to an integer, we try the following two methods:

1. If the function is linear, then we perform simple linear regression, round the resulting coefficients to integers, and simplify, e.g., multiplications by 0 and 1.
2. Otherwise, we use the brute force symbolic solver from *AI Feynman* [42], including the six unary operations {>,<,∼,H,D,A} and four binary operations {+,−,∗,%}, whose meanings are explained in Appendix C, then convert the simplest discovered formula into Python format.

**Figure 3:**
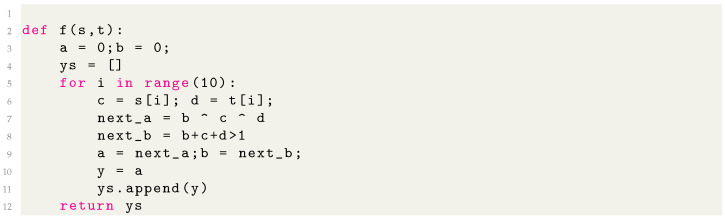
The generated program for the addition of two binary numbers represented as bit sequences. Note that MIPS rediscovers the “ripple adder”, where the variable *b* above is the carry bit.

**Figure 4:**
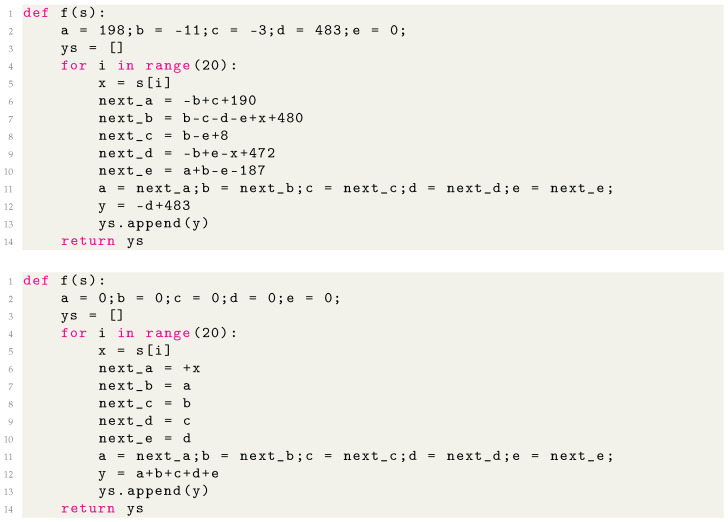
Comparison of code generated from an RNN trained on Sum_Last5, without (**top**) and with (**bottom**) normalizers.

Once symbolic formulas have been separately discovered for each component of the vector-valued functions *f* and *g*, we insert them into a template Python program that implements the basic loop over inputs that are inherent in an RNN. We present examples of our auto-generated programs in Figure 3 and Figure 4 and in Appendix G. Most hyperparameters are thresholds. For example, when a lattice has a reconstruction error ϵ below some threshold θ, we claim an integer lattice has been detected. Since we define the reconstruction errors to be dimensionless, it is clear that ϵ=1 means no structure is detected and ϵ=0 means perfect integer lattices. We find our results to be fairly robust with respect to θ; e.g., $\theta =10^{-2}-10^{-1}$ would yield the same results.

## 4. Results

We will now test the program synthesis abilities of our MIPS algorithm on a benchmark of algorithmic tasks specified by numerical examples. For comparison, we try the same benchmark on GPT-4 Turbo, which is currently (as of January 2024) described by OpenAI as their latest generation model, with a 128k context window and more capable than the original GPT-4.

### 4.1. Benchmark

Our benchmark consists of the 62 algorithmic tasks listed in Table 1. They each map one or two integer lists of length 10 or 20 into a new integer list. We refer to integers whose range is limited to {0,1} as bits. We generated this task list manually, attempting to produce a collection of diverse tasks that would in principle be solvable by an RNN. We also focused on tasks whose known algorithms involved majority, minimum, maximum, and absolute value functions because we believed they would be more easily learnable than other nonlinear algorithms due to our choice of the ReLU activation for our RNNs. The benchmark training data and project code are available at https://github.com/ejmichaud/neural-verification (accessed on 26 November 2024). The tasks are described in Table 1, with additional details in Appendix E. The benchmark aims to cover a diverse range of algorithmic tasks. To balance between different families, when a group of tasks is similar (e.g., summing up the last *k* bits), our convention is to keep (at most) six of them.

Since the focus of our paper is not on whether RNNs can learn algorithms, but on whether learned algorithms can be auto-extracted into Python, we discarded from our benchmark any generated tasks on which our RNN training failed to achieve 100% accuracy.

Our benchmark can never show that MIPS outperforms any large language model (LLM). Because LLMs are typically trained on GitHub, many LLMs can produce Python code for complicated programming tasks that fall outside of the class we study. Instead, the question that our MIPS-LLM comparison addresses is whether MIPS complements LLMs by being able to solve some tasks where an LLM fails.

### 4.2. Evaluation

**Figure 5:**
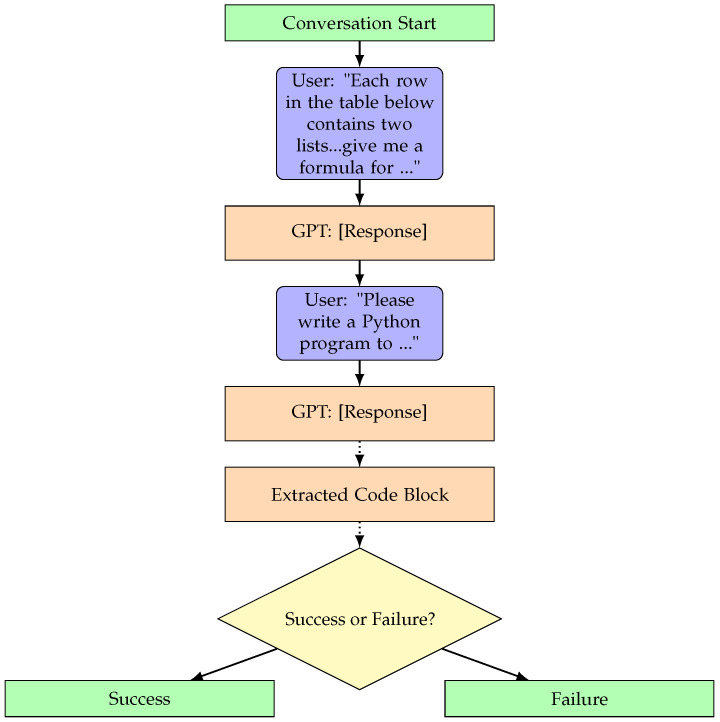
We compare MIPS against program synthesis with the large language model GPT-4 Turbo, prompted with a “chain-of-thought” approach. It begins with the user providing a task, followed by the model’s response, and culminates in assessing the success or failure of the generated Python code based on its accuracy in processing the provided lists.

For both our method and GPT-4 Turbo, a task is considered solved if and only if a Python program is produced that solves the task with 100% accuracy. GPT-4 Turbo is prompted using the “chain-of-thought” approach described below and illustrated in Figure 5.

For a given task, the LLM receives two lists of length 10 sourced from the respective RNN training set. The model is instructed to generate a formula that transforms the elements of list “x” (features) into the elements of list “y” (labels). Subsequently, the model is instructed to translate this formula into Python code. The model is specifically asked to use elements of the aforementioned lists as a test case and print “Success” or “Failure” if the generated function achieves full accuracy on the test case. An external program extracts a fenced markdown codeblock from the output, which is saved to a separate file and executed to determine if it successfully completes the task. To improve the chance of success, this GPT-4 Turbo prompting process is repeated three times, requiring only at least one of them to succeed. We run GPT using default temperature settings.

### 4.3. Performance

As seen in Table 1, MIPS is highly complementary to GPT-4 Turbo: MIPS solves 32 of our tasks, including 13 that are not solved by ChatGPT-4 (which solves 30).

The AutoML process of Section 3.1 discovers networks of varying task-dependent shape and size. Table A1 shows the parameters p discovered for each task. Across our 62 tasks, 16 tasks could be solved by a network with hidden dimensions n=1, and the largest *n* required was 81. For many tasks, there was an interpretable meaning to the shape of the smallest network we discovered. For instance, on tasks where the output is the element occurring *k* steps earlier in the list, we found n=k+1, since the current element and the previous *k* elements must be stored for later recall.

We found two main failure modes for MIPS:

1. Noise and non-linearity. The latent space is still close to being a finite state machine, but the non-linearity and/or noise present in an RNN is so dominant that the integer autoencoder fails, e.g., for *Diff_Abs_Values*. Humans can stare at the lookup table and regress the symbolic function with their brains, but since the lookup table is not perfect, i.e., it has the wrong integer in a few examples, MIPS fails to symbolically regress the function. This can probably be mitigated by learning and generalizing from a training subset with a smaller dynamic range.
2. Continuous computation. A key assumption of MIPS is that RNNs are finite-state machines. However, RNNs can also use continuous variables to represent information—the *Majority_0_X* tasks fail for this reason. This can probably be mitigated by identifying and implementing floating-point variables.

Figure 3 shows an example of a MIPS rediscovering the “ripple-carry adder” algorithm. The normalizers significantly simplified some of the resulting programs, as illustrated in Figure 4, and sometimes made the difference between MIPS failing and succeeding. We found that applying a small L1 weight regularization sometimes facilitated integer autoencoding by axis-aligning the lattice.

## 5. Discussion

We have presented MIPS, a novel method for program synthesis based on the automated mechanistic interpretability of neural networks trained to perform the desired task, auto-distilling the learned algorithm into Python code. Its essence is to first train a recurrent neural network to learn a clever finite state machine that performs the task and then automatically figure out how this machine works.

### 5.1. Findings

We found MIPS to be highly complementary to LLM-based program synthesis with GPT-4 Turbo, with each approach solving many tasks that stumped the other. Please note that our motivation is not to outcompete other program synthesis methods, but instead to provide a proof of principle that fully automated distillation of machine-learned algorithms is not impossible.

Whereas LLM-based methods have the advantage of drawing upon a vast corpus of human training data, MIPS has the advantage of discovering algorithms from scratch without human hints, with the potential to discover entirely new algorithms. As opposed to genetic programming approaches, MIPS leverages the power of deep learning by exploiting gradient information.

Program synthesis aside, our results shed further light on mechanistic interpretability, specifically on how neural networks represent bits and integers. We found that *n* integers tend to get encoded linearly in *n* dimensions, but generically in non-orthogonal directions with an additive offset. This is presumably because there are many more such messy encodings than simple ones, and the messiness can be easily (linearly) decoded. We saw that *n* bits sometimes get encoded as an *n*-dimensional parallelogram, but not always–––possibly because linear decodability is less helpful when the subsequent bit operations to be performed are nonlinear anyway.

### 5.2. Outlook

Our work is merely a modest first attempt at mechanistic-interpretability-based program synthesis, and there are many obvious generalizations worth trying in future work, for example,

1. Improvements in training and integer autoencoding (since many of our failed examples failed only just barely);
2. Generalization from RNNs to other architectures such as transformers;
3. Generalization from bits and integers to more general extractable data types such as floating-point numbers and various discrete mathematical structures and knowledge representations;
4. Scaling to tasks requiring much larger neural networks;
5. Automated formal verification of synthesized programs (we perform such verification with Dafny in Appendix G—Formal Verification to show that our MIPS-learned ripple adder correctly adds *any* binary numbers, not merely those in the test set, but such manual work should ideally be fully automated).

LLM-based coding co-pilots are already highly useful for program synthesis tasks based on verbal problem descriptions or auto-complete and will only get better. MIPS instead tackles program synthesis based on test cases alone. This makes it analogous to symbolic regression [42,43], which has already proven useful for various science and engineering applications [44,45] where one wishes to approximate data relationships with symbolic formulae. The MIPS framework generalizes symbolic regression from feed-forward formulae to programs with loops, which are in principle Turing-complete. If this approach can be scaled up, it may enable promising opportunities for making machine-learned algorithms more interpretable, verifiable, and trustworthy.

## Figures and Tables

**Table 1:** Benchmark results. For tasks with the note “see text”, please refer to Appendix E. The last column highlights the MIPS module responsible for each task. BR = boolean regression, LR = linear regression, SR = symbolic regression, and NA means MIPS is not expected to solve this problem. Green means success and red means failure.

Task #	Input Strings	Element Type	Task Description	Task Name	Solved by GPT-4?	Solved by MIPS?	MIPS Module
1	2	bit	Binary addition of two bit strings	Binary_Addition	0	1	BR
2	2	int	Ternary addition of two digit strings	Base_3_Addition	0	0	SR
3	2	int	Base 4 addition of two digit strings	Base_4_Addition	0	0	SR
4	2	int	Base 5 addition of two digit strings	Base_5_Addition	0	0	SR
5	2	int	Base 6 addition of two digit strings	Base_6_Addition	1	0	SR
6	2	int	Base 7 addition of two digit strings	Base_7_Addition	0	0	SR
7	2	bit	Bitwise XOR	Bitwise_Xor	1	1	BR
8	2	bit	Bitwise OR	Bitwise_Or	1	1	BR
9	2	bit	Bitwise AND	Bitwise_And	1	1	BR
10	1	bit	Bitwise NOT	Bitwise_Not	1	1	BR
11	1	bit	Parity of last 2 bits	Parity_Last2	1	1	BR
12	1	bit	Parity of last 3 bits	Parity_Last3	0	1	BR
13	1	bit	Parity of last 4 bits	Parity_Last4	0	0	BR
14	1	bit	Parity of all bits seen so far	Parity_All	0	1	BR
15	1	bit	Parity of number of zeros seen so far	Parity_Zeros	0	1	BR
16	1	int	Cumulative number of even numbers	Evens_Counter	0	0	BR
17	1	int	Cumulative sum	Sum_All	1	1	LR
18	1	int	Sum of last 2 numbers	Sum_Last2	0	1	LR
19	1	int	Sum of last 3 numbers	Sum_Last3	0	1	LR
20	1	int	Sum of last 4 numbers	Sum_Last4	1	1	LR
21	1	int	Sum of last 5 numbers	Sum_Last5	1	1	LR
22	1	int	sum of last 6 numbers	Sum_Last6	1	1	LR
23	1	int	Sum of last 7 numbers	Sum_Last7	1	1	LR
24	1	int	Current number	Current_Number	1	1	LR
25	1	int	Number 1 step back	Prev1	1	1	LR
26	1	int	Number 2 steps back	Prev2	1	1	LR
27	1	int	Number 3 steps back	Prev3	1	1	LR
28	1	int	Number 4 steps back	Prev4	1	1	LR
29	1	int	Number 5 steps back	Prev5	1	1	LR
30	1	int	1 if last two numbers are equal	Previous_Equals_Current	0	1	SR
31	1	int	current−previous	Diff_Last2	0	1	SR
32	1	int	|current−previous|	Abs_Diff	0	1	SR
33	1	int	|current|	Abs_Current	1	1	SR
34	1	int	|current|−|previous|	Diff_Abs_Values	1	0	SR
35	1	int	Minimum of numbers seen so far	Min_Seen	1	0	SR
36	1	int	Maximum of integers seen so far	Max_Seen	1	0	SR
37	1	int	integer in 0-1 with highest frequency	Majority_0_1	1	0	SR
38	1	int	Integer in 0-2 with highest frequency	Majority_0_2	0	0	SR
39	1	int	Integer in 0-3 with highest frequency	Majority_0_3	0	0	SR
40	1	int	1 if even, otherwise 0	Evens_Detector	1	0	SR
41	1	int	1 if perfect square, otherwise 0	Perfect_Square_Detector	0	0	SR
42	1	bit	1 if bit string seen so far is a palindrome	Bit_Palindrome	1	0	NA
43	1	bit	1 if parentheses balanced so far, else 0	Balanced_Parenthesis	0	0	SR
44	1	bit	Number of bits seen so far mod 2	Parity_Bits_Mod2	1	0	BR
45	1	bit	1 if last 3 bits alternate	Alternating_Last3	0	0	BR
46	1	bit	1 if last 4 bits alternate	Alternating_Last4	1	0	BR
47	1	bit	bit shift to right (same as prev1)	Bit_Shift_Right	1	1	LR
48	2	bit	Cumulative dot product of bits mod 2	Bit_Dot_Prod_Mod2	0	1	BR
49	1	bit	Binary division by 3 (see text)	Div_3	1	0	SR
50	1	bit	Binary division by 5 (see text)	Div_5	0	0	SR
51	1	bit	Binary division by 7 (see text)	Div_7	0	0	SR
52	1	int	Cumulative addition modulo 3	Add_Mod_3	1	1	SR
53	1	int	Cumulative addition modulo 4	Add_Mod_4	0	0	SR
54	1	int	Cumulative addition modulo 5	Add_Mod_5	0	0	SR
55	1	int	Cumulative addition modulo 6	Add_Mod_6	0	0	SR
56	1	int	Cumulative addition modulo 7	Add_Mod_7	0	0	SR
57	1	int	Cumulative addition modulo 8	Add_Mod_8	0	0	SR
58	1	int	1D dithering, 4-bit to 1-bit (see text)	Dithering	1	0	NA
59	1	int	Newton’s of - freebody (integer input)	Newton_Freebody	0	1	LR
60	1	int	Newton’s law of gravity (see text)	Newton_Gravity	0	1	LR
61	1	int	Newton’s law w. spring (see text)	Newton_Spring	0	1	LR
62	2	int	Newton’s law w. magnetic field (see text)	Newton_Magnetic	0	0	LR
			Total solved		30	32	

**Figure A1:**
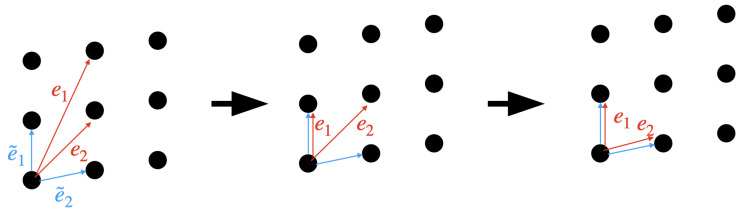
Both red and blue bases form a minimal parallelogram (in terms of cell volume) but one can further simplify red to blue by linear combination (simplicity in the sense of small $\ell_{2}$ norm).

## Data Availability

Code for reproducing our experiments can be found at https://github.com/ejmichaud/neural-verification (accessed on 26 November 2024).

## Appendix A. Lattice Finding Using a Generalized Greatest Common Divisor (Gcd)

Our method often encounters cases where hidden states secretly form an affine transformation of an integer lattice. However, not all lattice points are observed in training samples, so our goal is to recover the hidden integer lattice from sparse observations.

### Appendix A.1. Problem Formulation

Suppose we have a set of lattice points in $R^{D}$ spanned by *D* independent basis vectors, $b_{i}\;\left(i=1,2,\cdots ,D\right)$. Each lattice point *j* has the position

(A1) $$x_{j}=\sum_{i=1}^{D}a_{ji}b_{i}+c,$$

where c is a global translation vector, and the coefficients $a_{ji}$ are integers.

Our problem: given *N* such data points $\left(x_{1},x_{2},\cdots ,x_{N}\right)$, how can we recover the integer coefficients $a_{ji}$ for each point data point as well as $b_{i}$ and c?

Note that even when the whole lattice is given, there are still degrees of freedom for the solution. For example, $\left\{c\mapsto c+b_{i},a_{ji}\mapsto a_{ji}-1\right\}$ remains a solution, and $\left\{b_{i}\to \sum_{j=1}^{D}\Lambda_{ij}b_{j}\right\}$ remains a solution if Λ is an integer matrix whose determinant is ±1. So our success criteria are a s follows: (1) $a_{ji}$ are integers; (2) the discovered bases and the true bases have the same determinant (the volume of a unit cell). Once a set of bases is found, we can simplify them by minimizing their total norms over valid transformations ($\Lambda \in Z^{D\times D},\det\left(\Lambda \right)=\pm 1$).

### Appendix A.2. Regular GCD

As a reminder, given a list of *n* numbers $\left\{y_{1},y_{2},\cdots ,y_{n}\right\}$, a common divisor *d* is a number such that for all *i*, $\frac{y_{i}}{d}$ is an integer. All common divisors are the set $\{d|y_{i}/d\in Z$, and the greatest common divisor (GCD) is the largest number in this set. Because

(A2) $$\mathrm{GCD}\left(y_{1},\cdots ,y_{n}\right)=\mathrm{GCD}\left(y_{1},\mathrm{GCD}\left(y_{2},\mathrm{GCD}\left(y_{3},...\right)\right)\right),$$

it, without loss of generality, suffices to consider the case n=2. A common algorithm to compute GCD of two numbers is the so-called Euclidean algorithm. We start with two numbers $r_{0}$, $r_{1}$ and $r_{0}>r_{1}$, which is step 0. For the $k^{\mathrm{th}}$ step, we perform division-with-remainder to find the quotient $q_{k}$ and the remainder $r_{k}$ so that $r_{k-2}=q_{k}r_{k-1}+r_{k}$ with $|r_{k-1}\left|>\right|r_{k}|$ (We are considering a general case where $r_{0}$ and $r_{1}$ may be negative. Otherwise $r_{k}$ can always be positive numbers, hence no need to use the absolute function). The algorithm will eventually produce a zero remainder $r_{N}=0$, and the other non-zero remainder $r_{N-1}$ is the greatest common divisor. For example, GCD(55,45)=5, because

(A3) $$\begin{array}{cc} 55 & =1\times 45+10,\\ 45 & =4\times 10+5,\\ 10 & =2\times 5+0. \end{array}$$

### Appendix A.3. Generalized GCD in D Dimensions

Given a list of *n* vectors $\left\{y_{1},y_{2},\cdots ,y_{n}\right\}$ where $y_{i}\in R^{D}$, and assuming that these vectors are in the lattice described by Equation (A1), we can, without loss of generality, set c=0, since we can always redefine the origin. In *D* dimensions, the primitive of interest is the *D*-dimensional parallelogram: a line segment for D=1 (one basis vector), a parallelogram for D=2 (two basis vectors), parallelepiped for D=3 (three basis vectors), etc.

One can construct an *D*-dimensional parallelogram by constructing its basis vectors as a linear integer combination of $y_{j}$, i.e.,

(A4) $$q_{i}=\sum_{j=1}^{n}m_{ij}y_{j},m_{ij}\in Z,i=1,2,\cdots ,D.$$

The goal of *D*-dimensional GCD is to find a “minimal” parallelogram, such that its volume (which is $\det(q_{1},q_{2},\cdots ,q_{D})$) is the GCD of volumes of other possible parallelograms. Once the minimal parallelogram is found (There could be many minimal parallelograms, but finding one is sufficient), we can also determine $b_{i}$ in Equation (A1), since $b_{i}$ is exactly $q_{i}$! To find the minimal parallelogram, we need two steps: (1) figure out the unit volume; (2) figure out $q_{i}\left(i=1,2,\cdots \right)$ whose volume is the unit volume.

**Step 1: Compute unit volume $V_{0}$.** We first define *representative* parallelograms as one where all i=1,2,⋯,D, $m_{i}\equiv \left(m_{i1},m_{i2},\cdots ,m_{iD}\right)$ are one-hot vectors, i.e., with only one element being 1 and 0 otherwise. It is easy to show that the volume of any parallelogram is a linear integer combination of volumes of representative parallelograms, so in WLOG, we can focus on representative parallelograms. We compute the volumes of all representative parallelograms, which gives a volume array. Since volumes are just scalars, we can obtain the unit volume $V_{0}$ by calling the regular GCD of the volume array.

**Step 2: Find a minimal parallelogram (whose volume is the unit volume computed in step 1).** Recall that in regular GCD, we are dealing with two numbers (scalars). To leverage this in the vector case, we need to create scalars out of vectors and make sure that the vectors share the same linear structure as the scalars so that we can extend division and remainder to vectors. A natural scalar is volume. Now consider two parallelograms P1 and P2, which share D−1 basis vectors $\left(y_{3},\cdots ,y_{D+1}\right)$, but the last basis vector is different: $y_{1}$ for P1 and $y_{2}$ for P2. Denote their volume as $V_{1}$ and $V_{2}$:

(A5) $$\begin{array}{cc} V_{1} & =\det(y_{1},y_{3},y_{4},\cdots ,y_{D})\\ V_{2} & =\det(y_{2},y_{3},y_{4},\cdots ,y_{D}) \end{array}$$

Since

(A6) $$aV_{1}+bV_{2}=\det\left(ay_{1}+by_{2},y_{3},y_{4},\cdots ,y_{D}\right),$$

which shows that $\left(V_{1},V_{2}\right)$ and $\left(y_{1},y_{2}\right)$ share the same linear structure. We can simply apply division and remainder to $V_{1}$ and $V_{2}$ as in regular GCD:

(A7) $$V_{1}^{\prime },V_{2}^{\prime }=\mathrm{GCD}\left(V_{1},V_{2}\right),$$

whose quotients in all iterations are saved and transferred to $y_{1}$ and $y_{2}$:

(A8) $$y_{1}^{\prime },y_{2}^{\prime }=\mathrm{GCD}\_\mathrm{with}\_\mathrm{predefined}\_\mathrm{quotients}\left(y_{1},y_{2}\right).$$

If $V_{1}=V_{0}$ (which is the condition for minimal parallelogram), the algorithm terminates and returns $\left(y_{1}^{\prime },y_{3},y_{4},\cdots ,y_{D}\right)$. If $V_{1}>V_{0}$, we need to repeat step 2 with the new vector list $\left\{y_{1}^{\prime },y_{3},\cdots ,y_{N}\right\}$.

**Why can we remove $y_{2}^{\prime }$ for the next iteration?** Note that although eventually, $V_{1}^{\prime }>0$ and $V_{2}^{\prime }=0$, typically, $y_{2}\neq 0$. However, since

(A9) $$0=V_{2}^{\prime }=\det\left(y_{2}^{\prime },y_{3},y_{4},\cdots ,y_{D}\right),$$

this means $y_{2}^{\prime }$ is a linear combination of $\left(y_{3},\cdots ,y_{D}\right)$ and hence can be removed from the vector list.

**Step 3: Simplification of basis vectors.** We want to further simplify basis vectors. For example, the basis vectors obtained in step 2 may have large norms. For example, D=2, the standard integer lattice, has $b_{1}=\left(1,0\right)$ and $b_{2}=\left(0,1\right)$, but there are infinitely many possibilities after step 2, as long as pt−sq=±1 for $b_{1}=\left(p,q\right)$ and $b_{2}=\left(s,t\right)$, e.g., $b_{1}=\left(3,5\right)$ and $b_{2}=\left(4,7\right)$.

To minimize $\ell_{2}$ norms, we choose a basis and project and subtract for other bases. Note that (1) again, we are only allowed to subtract integer times of the chosen basis; (2) the volume of the parallelogram does not change since the project-and-subtract matrix has a determinant of 1 (suppose $b_{i}\left(i=2,3,\cdots ,D\right)$ are projected to $b_{1}$ and subtracted by multiples of $b_{1}$ and $p_{*}$ represents projection integers):

(A10) $$\left(\begin{array}{ccccc} 1 & p_{2\to 1} & p_{3\to 1} & \cdots & p_{D\to 1}\\ 0 & 1 & 0 & \cdots & 0\\ 0 & 0 & 1 & \cdots & 0\\ \vdots & \vdots & \vdots & \; & \vdots \\ 0 & 0 & 0 & \cdots & 1 \end{array}\right)$$

We do this iteratively until no norm can become shorter via the project-and-subtract procedure. Please see Figure A1 for an illustration of how simplification works for a 2D example.

**Computation overhead** is actually surprisingly small. In typical cases, we only need to call O(1) times of GCD.

**Dealing with noise**: Usually, the integer lattice in the hidden space is not perfect, i.e., vulnerable to noise. How do we extract integer lattices in a robust way in the presence of noise? Note that the terminating condition for the GCD algorithm is when the remainder is exactly zero–we relax this condition such that the absolute value of the remainder to be smaller than a threshold $\epsilon_{\gcd}$. Another issue regarding noise is that noise can accumulate in the GCD iterations, so we hope that GCD can converge in a few steps. To achieve this, we select hidden states in a small region with a data fraction p% of the whole data. Since both $\epsilon_{\gcd}$ and *p* depend on data and neural network training, which we do not know a priori, we choose to grid sweep $\epsilon_{\gcd}\in \left[10^{-3},1\right]$ and p∈(0.1,100); for each $\left(\epsilon_{\gcd},p\right)$, we obtain an integer lattice and compute its description length. We select the $\left(\epsilon_{\gcd},p\right)$ that gives the lattice with the smallest description length. The description length includes two parts: integer descriptions of hidden states $\log(1+|Z{|}^{2})$ and residual of reconstruction $\log(1+{\left(\frac{AZ+b-X}{\epsilon_{\mathrm{dl}}}\right)}^{2})$ with $\epsilon_{\mathrm{dl}}=10^{-4}$.

## Appendix B. Linear Lattice Finder

Although our RNN can represent general nonlinear functions, in the special case when the RNN actually performs linear functions, program synthesis can be much easier. So if the hidden MLP is linear, we would expect the hidden states to be an integer lattice, because inputs are integer lattices and the mappings are linear. Effectively, the hidden MLP works as a linear function: $h^{\left(t\right)}=W_{h}h^{\left(t-1\right)}+W_{i}x^{\left(t\right)}$ (we neglected the bias term since it is not relevant to finding basis vectors of a lattice).

Suppose we have input series $x^{\left(1\right)},x^{\left(2\right)},...,x^{\left(t\right)}$; then $h^{\left(t\right)}$ is

(A11) $$h^{\left(t\right)}=\sum_{j=1}^{t}W_{h}^{t-j}W_{i}x_{j},$$

Since $x_{j}$ values themselves are integer lattices, we could then interpret the following as basis vectors:

(A12) $$W_{h}^{t-j}W_{i},j=1,2,\cdots ,t,$$

which are not necessarily independent. For example, for the task of summing up the last two numbers, $W_{h}W_{i}$ and $W_{i}$ are non-zero vectors and are independent, while others $W_{h}^{n}W_{i}\approx 0,n\geq 2$. Then, $W_{h}W_{i}$ and $W_{i}$ are the two basis vectors for the lattice. In general, we measure the norm of all the candidate basis vectors and select the first *k* vectors with the highest norms, which are exactly basis vectors of the hidden lattice.

## Appendix C. Symbolic Regression

The formulation of symbolic regression is that one has a data pair $(x_{i},y_{i}),i=1,2,\cdots ,N$ with *N* data samples. The goal is to find a symbolic formula *f* such that $y_{i}=f\left(x_{i}\right)$. A function is expressed in reverse Polish notation (RPN); for example, |a|−c is expressed as aAc- where *A* stands for the absolute value function. We have three types of variables:

- type-0 operator. We include input variables and constants.
- type-1 operator (takes in one type-0 to produce one type-0). We include operations {>,<,∼,H,D,A}. > means +1; < means −1; ∼ means negating the number; *D* is dirac delta which outputs 1 only when taking in 0; *A* is the absolute value function;
- type-2 operator (takes in two type-0 to produce on type-0). We include operations {+,∗,−,%}. + means the addition of two numbers; ∗ means the multiplication of two numbers; − means the subtraction of two numbers; % is the remainder of one number module the other.

There are only certain types of templates (a string of numbers consisting of 0, 1, 2) that are syntactically correct. For example, 002 is correct while 02 is incorrect. We iterate over all the templates not longer than six symbols, and for each template, we try all the variable combinations. Each variable combination corresponds to a symbolic equation *f*, for which we can check whether $f\left(x_{i}\right)=y_{i}$ for 100 data points. If successful, we terminate the brute force program and return the successful formula. If a brute force search does not find any correct symbolic formula within the computing budget, we will simply return the formula *a*, to make sure that the program can still be synthesized but simply fail to make correct predictions.

## Appendix D. Neural Network Normalization Algorithms

It is well known that neural networks exhibit a large amount of symmetry. That is, there are many transformations that can be applied to networks without affecting the map y=f(x) that they compute. A classic example is to permute the neurons within layers.

In this section, we describe a suite of normalizers that we use to transform our networks into a standard form, such that the algorithms that they learn are easier to interpret. We call our five normalizers “Whitening”, “Jordan normal form (JNF)”, “Toeplitz”, “De-bias”, and “Quantization”.

The main symmetry that we focus on is a linear transformation of the hidden space h↦Ah, which requires the following changes to *f* and *g*:

$$\begin{array}{cc} f(h,x)=Wh+Vx+b\Rightarrow & f\left(h,x\right)=AWA^{-1}h+AVx+Ab\\ g(h)=G(Uh+c)\Rightarrow & f\left(h\right)=G\left(UA^{-1}h+c\right) \end{array}$$

and is implemented by changing the weights:

$$\begin{array}{cc} W\Rightarrow & AWA^{-1}\\ V\Rightarrow & AV\\ b\Rightarrow & Ab\\ U\Rightarrow & UA^{-1} \end{array}$$

For this symmetry, we can apply an arbitrary invertible similarity transformation A to W, which is the core idea underlying our normalizers, three of which have their own unique ways of constructing A, as we describe in the sections below. Most importantly, one of our normalizers exploits A to convert the hidden-to-hidden transformation W into a Jordan normal form, in the case where *f* is linear. Recent work has shown that large recurrent networks with linear hidden-to-hidden transformations, such as state space models [46], can perform just as well as transformer-based models in language modeling on a large scale. A major advantage of using linear hidden-to-hidden transformations is the possibility of expressing the hidden space in its eigenbasis. This causes the hidden-to-hidden transformation to become diagonal so that it can be computed more efficiently. In practice, modern state space models assume diagonality and go further to assume the elements on the diagonal are real; they fix the architecture to be this way during training.

By carrying this out, we ignore the possibility of linear hidden-to-hidden transformations that cannot be transformed into a real diagonal matrix via diagonalization. Such examples include rotation matrices (whose eigenvalues may be complex) and shift matrices (whose eigenvalues are degenerate and whose eigenvectors are duplicated). A more general form than the diagonal form is the Jordan normal form, which consists of Jordan blocks along the diagonal, each of which has the form λI+S for an eigenvalue λ, and the shift matrix S, with ones on the superdiagonal and zeros elsewhere. The diagonalization is a special case of the Jordan normal form, and all matrices can be transformed to the Jordan normal form. A simple transformation can also be applied to the Jordan normal forms that contain pairs of complex generalized eigenvectors to convert them into real matrices.

For nonlinear hidden-to-hidden transformations, we compute W as though the nonlinearities have been removed.

### Appendix D.1. Whitening Transformation

Similar to normalizing the means and variances of a dataset before feeding it into a machine learning model, a good first preprocessing step is to normalize the distribution of hidden states. We therefore choose to apply a whitening transformation to the hidden space. To compute the transformation, we compute the covariance matrix of hidden activations across the dataset and use the singular value decomposition (SVD) of this covariance matrix to find the closest transformation to the identity that will bring this covariance matrix to the identity. We ignore any directions with covariance less than ϵ=0.1, which cause more instability when normalized. We then post-apply this transformation to the last linear layer of the hidden-to-hidden transformation and its biases and pre-apply its inverse to the first layers of the hidden-to-hidden and hidden-to-output transformations. This leaves the net behavior of the network unchanged. Other transformations that we use in other normalizers operate in a similar manner by post-applying and pre-applying a transformation and its inverse transformation to the first and last layers that interact with the hidden space.

### Appendix D.2. Jordan Normal Form Transformation

Critically, the hidden-to-hidden transformations that we would like to convert into Jordan normal form are imperfect because they are learned. Eigenvectors belonging to each Jordan block must be identical, whereas this will only be approximately true of the learned transformation.

The Jordan normal form of a matrix is unstable; consider a matrix

$$\begin{array}{c} W=\left(\begin{array}{cc} 0 & 1\\ \delta & 0 \end{array}\right) \end{array}$$

which, when δ≠0, can be transformed into a Jordan normal form by

(A13) $$\begin{array}{c} \left(\begin{array}{cc} 0 & 1\\ \delta & 0 \end{array}\right)=\underset{T}{\underset{︸}{\left(\begin{array}{cc} 1 & 1\\ \sqrt{\delta } & -\sqrt{\delta } \end{array}\right)}}\left(\begin{array}{cc} \sqrt{\delta } & 0\\ 0 & -\sqrt{\delta } \end{array}\right){\left(\begin{array}{cc} 1 & 1\\ \sqrt{\delta } & -\sqrt{\delta } \end{array}\right)}^{-1} \end{array}$$

but when δ=0, is transformed into a Jordan normal form by

(A14) $$\begin{array}{c} \left(\begin{array}{cc} 0 & 1\\ 0 & 0 \end{array}\right)=\underset{T}{\underset{︸}{\left(\begin{array}{cc} 1 & 0\\ 0 & 1 \end{array}\right)}}\left(\begin{array}{cc} 0 & 1\\ 0 & 0 \end{array}\right){\left(\begin{array}{cc} 1 & 0\\ 0 & 1 \end{array}\right)}^{-1} \end{array}$$

As we can see, all of the matrices in the decomposition are unstable near δ=0, so the issue of error thresholding is not only numerical but is mathematical in nature as well.

We would like to construct an algorithm that computes the Jordan normal form with an error threshold |δ|<ϵ=0.7 within which the algorithm will pick the transformation T from Equation (A14) instead of from Equation (A13).

Our algorithm first computes the eigenvalues $\lambda_{i}$ and then iteratively solves for the generalized eigenvectors that lie in $\ker({\left(W-\lambda I\right)}^{k})$ for increasing *k*. The approximation occurs whenever we compute the kernel (of unknown dimension) of a matrix X; we take the SVD of X and treat any singular vectors as part of the nullspace if their singular values are lower than the threshold ϵ, calling the result ϵ-ker(X).

Spaces are always stored in the form of a rectangular matrix F of orthonormal vectors, and their dimension is always the width of the matrix. We build projections using $\mathrm{proj}\left(F\right)=FF^{H}$, where $F^{H}$ denotes the conjugate transpose of F. We compute kernels ker(X) of known dimension of matrices X by taking the SVD $X=V_{1}SV_{2}^{H}$ and taking the last singular vectors in $V_{2}^{H}$. We compute column spaces of projectors of known dimension by taking the top singular vectors of the SVD.

The steps in our algorithm are as follows:

1. Solve for the eigenvalues $\lambda_{i}$ of W, and check that eigenvalues that are within ϵ of each other form group, i.e., that $|\lambda_{i}-\lambda_{j}|\leq \epsilon$ and $|\lambda_{j}-\lambda_{k}|\leq \epsilon$ always imply $|\lambda_{k}-\lambda_{i}|\leq \epsilon$. Compute the mean eigenvalue for every group.
2. Solve for the approximate kernels of W−λI for each mean eigenvalue λ. We will denote this operation by ϵ-ker(W−λI). We represent these kernels by storing the singular vectors whose singular values are lower than ϵ. Also, we construct a “corrected matrix” of W−λI for every λ by taking the SVD, discarding the low singular values, and multiplying the pruned decomposition back together again.
3. Solve for successive spaces $F_{k}$ of generalized eigenvectors at increasing depths *k* along the set of Jordan chains with eigenvalue λ for all λ. In other words, find chains of mutually orthogonal vectors that are mapped to zero after exactly *k* applications of the map W−λI. We first solve for $F_{0}=\ker\left(W-\lambda I\right)$. Then for k>0, we first solve for $J_{k}=\epsilon \text{-}\ker\left(\left(I-\mathrm{proj}\left(F_{k-1}\right)\right)\left(W-\lambda I\right)\right)$ and deduce the number of chains which reach depth *k* from the dimension of $J_{k}$, and then solve for $F_{k}=\mathrm{col}\left(\mathrm{proj}\left(J_{k}\right)-\mathrm{proj}\left(F_{0}\right)\right)$.
4. Perform a consistency check to verify that the dimensions of $F_{k}$ always stay the same or decrease with *k*. Go through the spaces $F_{k}$ in reverse order, and whenever the dimension of $F_{k}$ decreases, figure out which direction(s) are not mapped to by applying W−λI to $F_{k+1}$. Carry this out by building a projector J from mapping vectors representing $F_{k+1}$ through W−λI and taking $\mathrm{col}(\mathrm{proj}\left(F_{k}\right)-J)$. Solve for the Jordan chain by repeatedly applying $\mathrm{proj}\left(F_{i}\right)\left(W_{i}-\lambda I\right)$ for *i* starting from k−1 and going all the way down to zero.
5. Concatenate all the Jordan chains together to form the transformation matrix T.

The transformation T consists of generalized eigenvectors that need not be completely real but may also include pairs of generalized eigenvectors that are complex conjugates of each other. Since we do not want the weights of our normalized network to be complex, we also apply a unitary transformation that changes any pair of complex generalized eigenvectors into a pair of real vectors and the resulting block of W into a multiple of a rotation matrix. As an example, for a real 2-by-2 matrix W with complex eigenvectors, we have

$$\begin{array}{cc} W & =T\left(\begin{array}{cc} a+\mathrm{bi} & 0\\ 0 & a-\mathrm{bi} \end{array}\right)T^{-1}\\ \; & =TT^{\prime }\left(\begin{array}{cc} a & -b\\ b & a \end{array}\right){\left(TT^{\prime }\right)}^{-1},\;T^{\prime }=\frac{1}{\sqrt{2}}\left(\begin{array}{cc} 1 & i\\ 1 & -i \end{array}\right) \end{array}$$

### Appendix D.3. Toeplitz Transformation

Once W is in Jordan-normal form, each Jordan block is an upper triangular Toeplitz matrix. Upper-triangular Toeplitz matrices, including Jordan blocks, will always commute with each other because they are all polynomials of the shift matrix (which has ones on the superdiagonal and zeros elsewhere), and therefore, these transformations will leave W unchanged but will still affect V. We split V up into parts operated on by each Jordan block and use these Toeplitz transformations to reduce the most numerically stable columns of each block of V to one-hot vectors. The numerical stability of a column vector is determined by the absolute value of the bottom element of that column vector, since its inverse will become the degenerate eigenvalues of the resulting Toeplitz matrix. If no column has a numerical stability above ϵ=0.0001, we pick the identity matrix for our Toeplitz transformation.

### Appendix D.4. De-Biasing Transformation

Oftentimes, W is not full-rank, and has a nontrivial nullspace. The bias b will have some component in the direction of this nullspace, and eliminating this component only affects the behavior of the output network *g*, and the perturbation cannot carry on to the remainder of the sequence via *f*. Therefore, we eliminate any such component and compensate accordingly by modifying the bias in the first affine layer of *g*. We identify the nullspaces by taking an SVD and identifying components whose singular value is less than ϵ=0.1.

### Appendix D.5. Quantization Transformation

After applying all of the previous transformations to the RNN, it is common for many of the weights to become close to zero or some other small integer. Treating this as a sign that the network is attempting to implement discrete operations using integers, we snap any weights and biases that are within a threshold ϵ=0.01 of an integer to that integer. For certain simple tasks, this sometimes allows the entire network to become quantized.

## Appendix E. Supplementary Training Data Details

Here, we present additional details on the benchmark tasks marked “see text” in Table 1:

- Div_3/5/7: This is a long division task for binary numbers. The input is a binary number, and the output is that binary number divided by 3, 5, or 7, respectively. The remainder is discarded. For example, we have 1000011/11 = 0010110 (67/3 = 22). The most significant bits occur first in the sequence.
- Dithering: This is a basic image color quantization task, for 1D images. We map 4-bit images to 1-bit images such that the cumulative sum of pixel brightnesses of both the original and dithered images remains as close as possible.
- Newton_Gravity: This is a Euler forward-propagation technique that follows the equation F=input−1,v↦v+F,x↦x+v.
- Newton_Spring: This is a Euler forward-propagation technique that follows the equation F=input−x,v↦v+F,x↦x+v.
- Newton_Magnetic: This is a Euler forward-propagation technique that follows the equation $F_{x}={\mathrm{input}}_{1}-v_{y},F_{y}={\mathrm{input}}_{2}+v_{x},v\mapsto v+F,x\mapsto x+v$.

## Appendix F. Architecture Search Results

Table A1 shows the parameters p discovered for each task by our architecture search.

**Table A1:** AutoML architecture search results. All networks achieved 100% accuracy on at least one test batch.

Task #	Task Name	*n*	$w_{f}$	$d_{f}$	$w_{g}$	$d_{g}$	Train Loss	Test Loss
1	Binary_Addition	2	4	2	–	1	0	0
2	Base_3_Addition	2	5	2	–	1	0	0
3	Base_4_Addition	2	5	2	–	1	0	0
4	Base_5_Addition	2	5	2	–	1	0	0
5	Base_6_Addition	2	6	2	–	1	2.45 × 10^−9^	2.53 × 10^−9^
6	Base_7_Addition	2	10	2	–	1	2.32 × 10^−6^	2.31 × 10^−6^
7	Bitwise_Xor	1	2	2	–	1	0	0
8	Bitwise_Or	1	–	1	–	1	3.03 × 10^−2^	3.03 × 10^−2^
9	Bitwise_And	1	–	1	–	1	3.03 × 10^−2^	3.03 × 10^−2^
10	Bitwise_Not	1	–	1	–	1	0	0
11	Parity_Last2	1	229	2	–	1	1.68 × 10^−2^	1.69 × 10^−2^
12	Parity_Last3	2	5	2	–	1	1.62 × 10^−4^	1.64 × 10^−4^
13	Parity_Last4	3	29	2	–	1	3.07 × 10^−7^	2.99 × 10^−7^
14	Parity_All	1	2	2	–	1	0	0
15	Parity_Zeros	1	2	2	–	1	0	0
16	Evens_Counter	4	73	3	–	1	8.89 × 10^−5^	8.88 × 10^−5^
17	Sum_All	1	–	1	–	1	6.09 × 10^−8^	6.13 × 10^−8^
18	Sum_Last2	2	–	1	–	1	0	0
19	Sum_Last3	3	–	1	–	1	6.34 × 10^−7^	6.35 × 10^−7^
20	Sum_Last4	4	–	1	–	1	2.10 × 10^−4^	2.11 × 10^−4^
21	Sum_Last5	5	–	1	–	1	8.86 × 10^−3^	8.87 × 10^−3^
22	Sum_Last6	6	–	1	–	1	1.82 × 10^−2^	1.81 × 10^−2^
23	Sum_Last7	7	–	1	–	1	3.03 × 10^−2^	3.01 × 10^−2^
24	Current_Number	1	–	1	–	1	0	0
25	Prev1	2	–	1	–	1	0	0
26	Prev2	3	–	1	–	1	0	0
27	Prev3	4	–	1	–	1	0	0
28	Prev4	5	–	1	–	1	2.04 × 10^−7^	2.05 × 10^−7^
29	Prev5	6	–	1	–	1	6.00 × 10^−5^	5.96 × 10^−5^
30	Previous_Equals_Current	2	5	2	–	1	6.72 × 10^−5^	6.61 × 10^−5^
31	Diff_Last2	2	–	1	–	1	0	0
32	Abs_Diff	2	–	1	2	2	1.84 × 10^−7^	1.84 × 10^−7^
33	Abs_Current	1	2	2	–	1	4.51 × 10^−8^	5.71 × 10^−8^
34	Diff_Abs_Values	2	4	2	–	1	3.15 × 10^−6^	2.96 × 10^−6^
35	Min_Seen	1	2	2	–	1	0	0
36	Max_Seen	1	2	2	–	1	1.46 × 10^−12^	0
37	Majority_0_1	1	63	2	–	1	4.03 × 10^−3^	4.05 × 10^−3^
38	Majority_0_2	4	98	2	–	1	1.64 × 10^−4^	1.71 × 10^−4^
39	Majority_0_3	21	132	3	–	1	6.94 × 10^−5^	6.86 × 10^−5^
40	Evens_Detector	5	163	2	–	1	8.18 × 10^−4^	8.32 × 10^−4^
41	Perfect_Square_Detector	48	100	2	–	1	1.92 × 10^−3^	1.97 × 10^−3^
42	Bit_Palindrome	18	86	2	–	1	3.81 × 10^−5^	3.69 × 10^−5^
43	Balanced_Parenthesis	1	16	2	–	1	7.44 × 10^−3^	7.10 × 10^−3^
44	Parity_Bits_Mod2	1	–	1	–	1	0	0
45	Alternating_Last3	2	3	2	–	1	1.85 × 10^−2^	1.87 × 10^−2^
46	Alternating_Last4	2	3	2	–	1	8.24 × 10^−6^	8.09 × 10^−6^
47	Bit_Shift_Right	2	–	1	–	1	0	0
48	Bit_Dot_Prod_Mod2	1	3	2	–	1	0	0
49	Div_3	2	59	2	–	1	6.40 × 10^−3^	6.43 × 10^−3^
50	Div_5	4	76	2	–	1	1.50 × 10^−4^	1.55 × 10^−4^
51	Div_7	4	103	2	–	1	6.65 × 10^−4^	6.63 × 10^−4^
52	Add_Mod_3	1	149	2	–	1	1.02 × 10^−3^	1.04 × 10^−3^
53	Add_Mod_4	2	33	2	–	1	1.53 × 10^−4^	1.44 × 10^−4^
54	Add_Mod_5	3	43	2	–	1	1.02 × 10^−3^	1.03 × 10^−3^
55	Add_Mod_6	4	108	2	–	1	6.14 × 10^−4^	6.12 × 10^−4^
56	Add_Mod_7	4	199	2	–	1	3.96 × 10^−4^	4.07 × 10^−4^
57	Add_Mod_8	67	134	2	–	1	8.53 × 10^−4^	8.34 × 10^−4^
58	Dithering	81	166	2	–	1	7.72 × 10^−4^	7.75 × 10^−4^
59	Newton_Freebody	2	–	1	–	1	2.61 × 10^−7^	2.62 × 10^−7^
60	Newton_Gravity	2	–	1	–	1	1.81 × 10^−7^	1.87 × 10^−7^
61	Newton_Spring	2	–	1	–	1	0	0
62	Newton_Magnetic	4	–	1	–	1	8.59 × 10^−5^	8.60 × 10^−5^

## Appendix G. Generated Programs

This section includes all successfully generated Python programs.

Binary-Addition

Bitwise-Xor

Bitwise-Or

Bitwise-And

Bitwise-Not

Parity-Last2

Parity-Last3

Parity-All

Parity-Zeros

Sum-All

Sum-Last2

Sum-Last3

Sum-Last4

Sum-Last5

Sum-Last6

Sum-Last7

Current-Number

Prev1

Prev2

Prev3

Prev4

Prev5

Previous-Equals-Current

Diff-Last2

Abs-Diff

Abs-Current

Bit-Shift-Right

Bit-Dot-Prod-Mod2

Add-Mod-3

Newton-Freebody

Newton-Gravity

Newton-Spring

### Formal Verification

The Dafny programming language is designed so that programs can be formally verified for correctness. The desired behavior of a program can be explicitly specified via preconditions, postconditions, and invariants, which are verified via automated theorem proving. These capabilities make Dafny useful in fields where correctness and safety are crucial.

We leverage Dafny’s robust verification capabilities to prove the correctness of the bit addition Python program synthesized by MIPS. The bit addition Python program was first converted to Dafny, then annotated with specific assertions, preconditions, and postconditions that defined the expected behavior of the code. Each annotation in the code was then formally verified by Dafny, ensuring that under all possible valid inputs, the code’s output would be consistent with the expected behavior. On line 79, we show that the algorithm found by MIPS is indeed equivalent to performing bit addition with length 10 bitvectors in Dafny.

Dafny-Code

## References

[1] Center for AI Safety (2023). Statement on AI Risk. https://www.safe.ai/work/statement-on-ai-risk.

[2] Tegmark M., Omohundro S. (2023). Provably safe systems: The only path to controllable agi. arXiv.

[3] Dalrymple D., Skalse J., Bengio Y., Russell S., Tegmark M., Seshia S., Omohundro S., Szegedy C., Goldhaber B., Ammann N. (2024). Towards Guaranteed Safe AI: A Framework for Ensuring Robust and Reliable AI Systems. arXiv.

[4] Zhou B., Ding G. (2023). Survey of intelligent program synthesis techniques. Proceedings of the International Conference on Algorithms, High Performance Computing, and Artificial Intelligence (AHPCAI 2023).

[5] Odena A., Shi K., Bieber D., Singh R., Sutton C., Dai H. (2020). BUSTLE: Bottom-Up program synthesis through learning-guided exploration. arXiv.

[6] Wu J., Wei L., Jiang Y., Cheung S.C., Ren L., Xu C. (2023). Programming by Example Made Easy. ACM Trans. Softw. Eng. Methodol..

[7] Sobania D., Briesch M., Rothlauf F. Choose your programming copilot: A comparison of the program synthesis performance of github copilot and genetic programming. Proceedings of the Genetic and Evolutionary Computation Conference.

[8] Olah C., Cammarata N., Schubert L., Goh G., Petrov M., Carter S. (2020). Zoom in: An introduction to circuits. Distill.

[9] Cammarata N., Goh G., Carter S., Schubert L., Petrov M., Olah C. (2020). Curve Detectors. Distill.

[10] Wang K., Variengien A., Conmy A., Shlegeris B., Steinhardt J. (2022). Interpretability in the wild: A circuit for indirect object identification in gpt-2 small. arXiv.

[11] Olsson C., Elhage N., Nanda N., Joseph N., DasSarma N., Henighan T., Mann B., Askell A., Bai Y., Chen A. (2022). In-context Learning and Induction Heads. Transform. Circuits Thread.

[12] Goh G., Cammarata N., Voss C., Carter S., Petrov M., Schubert L., Radford A., Olah C. (2021). Multimodal Neurons in Artificial Neural Networks. Distill.

[13] Gurnee W., Tegmark M. (2023). Language models represent space and time. arXiv.

[14] Vafa K., Chen J.Y., Kleinberg J., Mullainathan S., Rambachan A. (2024). Evaluating the World Model Implicit in a Generative Model. arXiv.

[15] Burns C., Ye H., Klein D., Steinhardt J. (2022). Discovering latent knowledge in language models without supervision. arXiv.

[16] Marks S., Tegmark M. (2023). The geometry of truth: Emergent linear structure in large language model representations of true/false datasets. arXiv.

[17] McGrath T., Kapishnikov A., Tomavsev N., Pearce A., Wattenberg M., Hassabis D., Kim B., Paquet U., Kramnik V. (2022). Acquisition of chess knowledge in alphazero. Proc. Natl. Acad. Sci. USA.

[18] Toshniwal S., Wiseman S., Livescu K., Gimpel K. Chess as a testbed for language model state tracking. Proceedings of the AAAI Conference on Artificial Intelligence.

[19] Li K., Hopkins A.K., Bau D., Viégas F., Pfister H., Wattenberg M. (2022). Emergent world representations: Exploring a sequence model trained on a synthetic task. arXiv.

[20] Nanda N., Chan L., Liberum T., Smith J., Steinhardt J. (2023). Progress measures for grokking via mechanistic interpretability. arXiv.

[21] Liu Z., Kitouni O., Nolte N., Michaud E.J., Tegmark M., Williams M. Towards Understanding Grokking: An Effective Theory of Representation Learning. Proceedings of the Thirty-Sixth Conference on Neural Information Processing Systems.

[22] Zhong Z., Liu Z., Tegmark M., Andreas J. The clock and the pizza: Two stories in mechanistic explanation of neural networks. Advances in Neural Information Processing Systems: 37th Conference on Neural Information Processing Systems (NeurIPS 2023), New Orleans, LA, USA, 10–16 December 2023.

[23] Quirke P., Barez F. (2023). Understanding Addition in Transformers. arXiv.

[24] Chughtai B., Chan L., Nanda N., Krause A., Brunskill E., Cho K., Engelhardt B., Sabato S., Scarlett J. A Toy Model of Universality: Reverse Engineering how Networks Learn Group Operations. Proceedings of the 40th International Conference on Machine Learning.

[25] Hanna M., Liu O., Variengien A. (2023). How does GPT-2 compute greater-than?: Interpreting mathematical abilities in a pre-trained language model. arXiv.

[26] Charton F. (2023). Can transformers learn the greatest common divisor?. arXiv.

[27] Lindner D., Kramár J., Farquhar S., Rahtz M., McGrath T., Mikulik V. (2023). Tracr: Compiled transformers as a laboratory for interpretability. arXiv.

[28] Friedman D., Wettig A., Chen D. (2023). Learning transformer programs. Adv. Neural Inf. Process. Syst..

[29] Bills S., Cammarata N., Mossing D., Tillman H., Gao L., Goh G., Sutskever I., Leike J., Wu J., Saunders W. (2023). Language Models Can Explain Neurons in Language Models. https://openaipublic.blob.core.windows.net/neuron-explainer/paper/index.html.

[30] Cunningham H., Ewart A., Riggs L., Huben R., Sharkey L. (2023). Sparse autoencoders find highly interpretable features in language models. arXiv.

[31] Bricken T., Templeton A., Batson J., Chen B., Jermyn A., Conerly T., Turner N., Anil C., Denison C., Askell A. (2023). Towards Monosemanticity: Decomposing Language Models with Dictionary Learning. Transform. Circuits Thread.

[32] Conmy A., Mavor-Parker A.N., Lynch A., Heimersheim S., Garriga-Alonso A. (2023). Towards automated circuit discovery for mechanistic interpretability. arXiv.

[33] Syed A., Rager C., Conmy A. (2023). Attribution Patching Outperforms Automated Circuit Discovery. arXiv.

[34] Marks S., Rager C., Michaud E.J., Belinkov Y., Bau D., Mueller A. (2024). Sparse feature circuits: Discovering and editing interpretable causal graphs in language models. arXiv.

[35] Karpathy A., Johnson J., Fei-Fei L. (2015). Visualizing and understanding recurrent networks. arXiv.

[36] Strobelt H., Gehrmann S., Pfister H., Rush A.M. (2018). LSTMVis: A Tool for Visual Analysis of Hidden State Dynamics in Recurrent Neural Networks. IEEE Trans. Vis. Comput. Graph..

[37] Giles C.L., Horne B.G., Lin T. (1995). Learning a class of large finite state machines with a recurrent neural network. Neural Netw..

[38] Wang Q., Zhang K., Ororbia II A.G., Xing X., Liu X., Giles C.L. (2017). An empirical evaluation of rule extraction from recurrent neural networks. arXiv.

[39] Weiss G., Goldberg Y., Yahav E. Extracting automata from recurrent neural networks using queries and counterexamples. Proceedings of the 35th International Conference on Machine Learning.

[40] Oliva C., Lago-Fernández L.F. (2019). On the interpretation of recurrent neural networks as finite state machines. Proceedings of the Artificial Neural Networks and Machine Learning–ICANN 2019: Theoretical Neural Computation: 28th International Conference on Artificial Neural Networks.

[41] Muvskardin E., Aichernig B.K., Pill I., Tappler M. (2022). Learning finite state models from recurrent neural networks. Proceedings of the International Conference on Integrated Formal Methods.

[42] Udrescu S.M., Tan A., Feng J., Neto O., Wu T., Tegmark M. (2020). AI Feynman 2.0: Pareto-optimal symbolic regression exploiting graph modularity. Adv. Neural Inf. Process. Syst..

[43] Cranmer M. (2023). Interpretable machine learning for science with PySR and SymbolicRegression. jl. arXiv.

[44] Cranmer M., Sanchez Gonzalez A., Battaglia P., Xu R., Cranmer K., Spergel D., Ho S. (2020). Discovering symbolic models from deep learning with inductive biases. Adv. Neural Inf. Process. Syst..

[45] Ma H., Narayanaswamy A., Riley P., Li L. (2022). Evolving symbolic density functionals. Sci. Adv..

[46] Gu A., Dao T. (2023). Mamba: Linear-time sequence modeling with selective state spaces. arXiv.

